# Investigation of the Relationship Between Glycemic Control and Inflammation–Nutrition Indices in Older Adults with Type 2 Diabetes

**DOI:** 10.3390/medicina62020369

**Published:** 2026-02-12

**Authors:** Feyza Mutlay, Murat Das, Merve Durmaz Yıldız, Ferhan Demirer Aydemir, Ece Ünal Çetin, Özge Kurtkulağı

**Affiliations:** 1 Division of Geriatrics, Department of Internal Medicine, Faculty of Medicine, Canakkale Onsekiz Mart University, Canakkale 17020, Turkey; 2 Department of Emergency Medicine, Faculty of Medicine, Canakkale Onsekiz Mart University, Canakkale 17020, Turkey; muratdas58@gmail.com; 3 Department of Internal Medicine, Faculty of Medicine, Canakkale Onsekiz Mart University, Canakkale 17020, Turkey; durmazzmerve16@gmail.com (M.D.Y.); eceunalcetin@gmail.com (E.Ü.Ç.); ozgekurtkulagi@gmail.com (Ö.K.); 4 Intensive Care Unit, Department of Internal Medicine, Faculty of Medicine, Canakkale Onsekiz Mart University, Canakkale 17020, Turkey; ferhanaydemirer@gmail.com

**Keywords:** older adults, HALP score, EASIX score, uric acid-to-HDL cholesterol ratio, 30-day mortality

## Abstract

*Objective*: To investigate the relationship between glycemic control and inflammation–nutrition indices in older adults with type 2 diabetes mellitus and to evaluate their prognostic value for 30-day mortality. *Methods*: This retrospective cohort study included 372 hospitalized patients aged ≥65 years with type 2 diabetes. Laboratory data were used to calculate the hemoglobin–albumin–lymphocyte–platelet (HALP) score, the endothelial activation and stress index (EASIX), and the uric acid-to-high-density lipoprotein cholesterol ratio (UHR). Cox regression analyses were performed to identify independent predictors of 30-day mortality, and combined stratification models using HALP, EASIX, and UHR were evaluated for risk discrimination. *Results*: Thirty-day mortality occurred in 57 patients (15.3%). HbA1c levels were not significantly associated with mortality (*p* = 0.615). Non-survivors had higher UHR, and EASIX, and lower HALP score levels (all *p* < 0.05). In multivariate Cox regression, age (HR 1.066, 95% CI 1.024–1.109, *p* = 0.002), length of hospital stay (HR 1.050, 95% CI 1.026–1.074, *p* < 0.001), ICU admission (HR 2.394, 95% CI 1.227–4.672, *p* = 0.010), and UHR (HR 1.028, 95% CI 1.013–1.042, *p* < 0.001) were independent predictors of mortality. Stratification by EASIX and UHR revealed that patients with both high EASIX or UHR and low HALP had the highest mortality risk, with adjusted HRs up to 4.206 (95% CI 1.930–9.166, *p* < 0.001). *Conclusions*: Among older adults with type 2 diabetes, short-term mortality is more strongly associated with inflammation, endothelial stress, and nutritional status than with glycemic control. Combined inflammation–nutrition indices (HALP, EASIX, UHR) provide superior risk stratification and help identify high-risk patients early.

## 1. Introduction

Type 2 diabetes (T2DM) is a significant public health problem worldwide, and its prevalence is increasing dramatically among older adults [1]. Age-related physiological changes, multiple diseases, and multiple medication use make older adults with diabetes vulnerable to adverse clinical outcomes, including increased hospitalizations and short-term mortality [2]. Although optimal glycemic control is a cornerstone of diabetes management, the prognostic value of traditional glycemic markers in older adult patients with acute illness remains controversial.

Chronic low-grade inflammation is a key component of the pathophysiology of T2DM and becomes more pronounced with age. This persistent inflammatory state contributes to insulin resistance, endothelial dysfunction, and accelerated atherosclerosis, increasing susceptibility to acute illness and poor outcomes [3]. In parallel, older adults with diabetes are at risk of malnutrition due to reduced dietary intake, sarcopenia, chronic inflammation, and metabolic dysregulation [4]. The combination of inflammation and malnutrition creates a vicious cycle that can significantly impair immune function and physiological resistance during hospitalization.

In recent years, various inflammation- and nutrition-based indices derived from routine laboratory parameters have emerged as practical tools for risk assessment. The hemoglobin–albumin–lymphocyte–platelet (HALP) score reflects both nutritional and immunological status, while the endothelial activation and stress index (EASIX) serves as an indicator of endothelial dysfunction and systemic stress [5,6]. The uric acid–high-density lipoprotein cholesterol ratio (UHR) integrates metabolic and inflammatory components and has been linked to adverse cardiovascular and metabolic outcomes [7]. Although each index has prognostic potential, the combined value of these indices in older adults with type 2 diabetes has not been sufficiently investigated.

Furthermore, the relationship between glycemic control and inflammatory–nutritional indices in older adult diabetic patients remains unclear [8,9]. Although hemoglobin A1c (HbA1c) is commonly used to assess long-term glycemic exposure, it may not accurately reflect acute metabolic stress, inflammatory burden, or nutritional impairment during hospitalization. Consequently, there is a need to identify complementary markers that better reflect short-term risk and clinical vulnerability in this population. Healthcare professionals can readily calculate HALP scores, EASIX scores, and uric acid/HDL–cholesterol (UHR) ratios using standard laboratory parameters that are routinely collected in daily practice, without incurring extra costs or tests. In clinical environments, these models assist in identifying patients with poor glycemic control who may require closer monitoring, nutritional support, lifestyle modifications, or a multidisciplinary approach.

Therefore, this study aimed to investigate the relationship between glycemic control and inflammatory–nutritional indices in older adults with type 2 diabetes and to evaluate their prognostic significance for 30-day mortality. We also aimed to determine whether combining HALP, EASIX, and UHR could improve risk discrimination beyond traditional glycemic markers.

## 2. Materials and Methods

### 2.1. Study Design and Participants

This study uses a retrospective observational design to examine a cohort of patients aged 65 and older with type 2 diabetes who were admitted to and treated at our internal medicine clinic between April 2020 and November 2025. The cohort of 372 individuals was divided into two subgroups based on good and poor glycemic control. The study was conducted in accordance with the Declaration of Helsinki and approved by the institution’s ethics committee (approval date: 12 November 2025; committee decision number: 2025-16/16-10).

### 2.2. Sample Size

**Figure 1:**
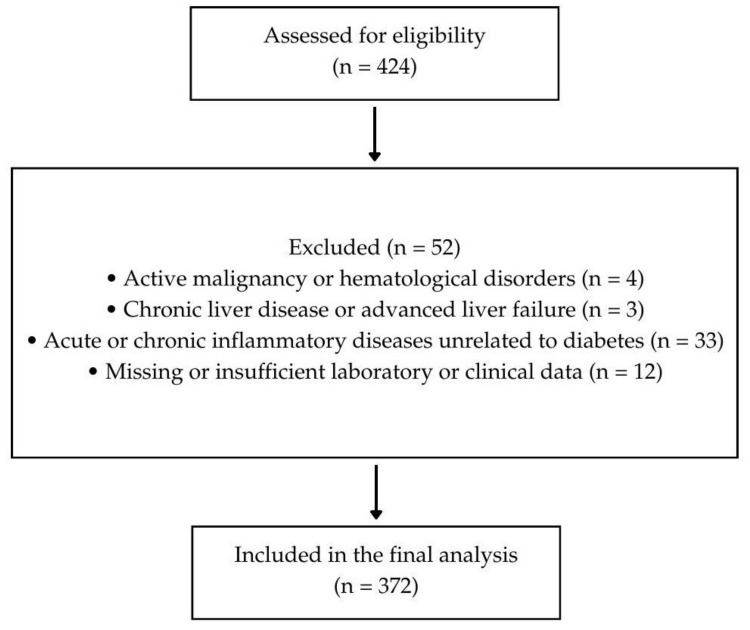
Participant flow diagram showing the screening, exclusion criteria, and final inclusion of older adults with type 2 diabetes in the study.

The sample size was determined based on previously published data evaluating the prognostic value of the HALP score in diabetic patients. Erbay and Aladağ (2025) [10] reported that patients with lower HALP scores had higher rates of adverse cardiovascular outcomes (29.6% in the high-risk group vs. 13.7% in the low-risk group). Using these proportions, a two-sided alpha of 0.05 and a power of 80%, the minimum required sample size was approximately 180 patients. To ensure adequate power for multivariate analyses, a total of 372 patients were included (Figure 1).

#### 2.2.1. Inclusion Criteria

Patients were eligible for inclusion in the study if they met all of the following criteria:

– Age ≥ 65 years at hospital admission.
– Diagnosis of type 2 diabetes mellitus according to American Diabetes Association criteria.
– Availability of complete laboratory data required to calculate inflammation–nutrition indices, including HALP, EASIX, and UHR.

#### 2.2.2. Exclusion Criteria

Patients were excluded from the study if they met any of the following criteria:

– Diagnosis of type 1 diabetes mellitus or secondary forms of diabetes.
– Active malignancy or hematological disorders affecting hemoglobin, platelet, or lymphocyte counts.
– Chronic liver disease or advanced liver failure.
– Acute or chronic inflammatory diseases unrelated to diabetes that may affect inflammation–nutrition indices (e.g., sepsis unrelated to diabetes, autoimmune disorders).
– Missing or insufficient laboratory or clinical data required for study analyses.
– Hospitalization due to trauma or planned surgery.

### 2.3. Data Collection

Demographic, clinical, and laboratory data were obtained from electronic medical records. Comorbidities, including hypertension, coronary artery disease, chronic renal failure, and stroke history, were recorded. Vital signs, length of hospital stay, and admission to the intensive care unit (ICU) were documented. The number of chronic medications was also recorded to assess polypharmacy.

### 2.4. Laboratory Measurements

Retrospective data collection was conducted using the hospital’s electronic medical records. Blood samples were drawn at admission as part of routine care and analyzed for hemoglobin, albumin, lymphocyte count, platelet count, creatinine, uric acid, glucose, lipid profile, C-reactive protein (CRP), ferritin, vitamin B12, and 25-hydroxyvitamin D levels. Routine biochemical tests, including glucose, lipid profile, uric acid, creatinine, lactate dehydrogenase (LDH), and vitamin D, were performed with an automated chemistry analyzer (Roche Cobas c702, Roche Diagnostics, Mannheim, Germany). Complete blood count parameters, such as hemoglobin, lymphocytes, and platelets, were measured using an automated hematology analyzer (Mindray BC-6000, Mindray Bio-Medical Electronics, Shenzhen, China). Serum albumin was assessed via standard automated colorimetric methods. Hemoglobin A1c (HbA1c) was determined through high-performance liquid chromatography (HPLC) using a dedicated analyzer (Lifotronic H9, Lifotronic Technology, Shenzhen, China). The estimated glomerular filtration rate (eGFR) was calculated using the CKD-EPI formula. An HbA1c below 7.5% indicated adequate glycemic control, whereas a level of 7.5% or higher signified poor control. All tests were conducted in the hospital’s central laboratory according to manufacturer instructions and quality control standards.

### 2.5. Inflammation–Nutrition Indices

The following indices were calculated:

- HALP score = hemoglobin (g/L) × albumin (g/L) × lymphocyte count (/L) ÷ platelet count (/L) [11].
- Endothelial Activation and Stress Index (EASIX) = [formula based on LDH × creatinine/platelet count] [6].
- Uric Acid-to-HDL Cholesterol Ratio (UHR) = uric acid (mg/dL) ÷ HDL cholesterol (mg/dL) [12].

### 2.6. Statistical Analysis

In this study, the data were analyzed using the IBM SPSS 29.0 software package (IBM Corp., Armonk, NY, USA). Categorical frequencies (n) and percentages (%) were reported, and continuous variables were presented as mean ± standard deviation (SD) or median (min-max). The normality of the distribution of continuous variables was assessed using the Shapiro–Wilk test. The Mann–Whitney U test was used to determine whether there was a statistically significant difference between groups. The Chi-square test was applied to categorical parameters.

The relationships between clinical, laboratory, and composite indices (HALP, EASIX, UHR) and 30-day mortality were evaluated using univariate and multivariate Cox proportional hazards regression, and hazard ratios (HR) and 95% confidence intervals (CI) were reported. Variables with *p* < 0.10 in univariate analyses were included in multivariate models.

To reduce potential bias inherent in the retrospective study design, we applied predefined inclusion and exclusion criteria consistently, including all consecutive eligible patients admitted during the study period. Laboratory parameters used for index calculations were collected at admission, prior to outcome occurrence, to minimize measurement bias. Patients lacking sufficient data for index calculation or outcome assessment were excluded, and a complete-case analysis was conducted without data imputation. For stratified analyses, patients were divided into high- and low-risk groups for EASIX, HALP, and UHR based on the median values of each index within the study population. This approach prevented overfitting and helped maintain balanced group sizes. A *p* value < 0.05 (two-sided) was deemed statistically significant.

## 3. Results

### 3.1. Key Characteristics of the Participants

Among the 372 diabetic patients included in the study, the most common primary reason for hospitalization was renal and electrolyte disorders (27.7%), followed by endocrine and metabolic disorders (22.3%) and anemia-related disorders (18.8%). Infectious diseases accounted for 13.2% of hospitalizations, while gastrointestinal, cardiovascular, and respiratory disorders were less frequent, representing 8.1%, 6.5%, and 3.5% of hospitalizations, respectively (Table 1).

### 3.2. The Relationship Between HALP, EASIX, and UHR Scores and Glycemic Control

As shown in Table 2, patients with HbA1c ≥ 7.5% had significantly higher HALP scores than patients with HbA1c < 7.5% (median [IQR]: 26.87 [14.75–42.58] vs. 18.58 [11.81–31.55], *p* < 0.001). In contrast, no significant difference was observed between the two groups in EASIX and UHR scores (*p* = 0.566 and *p* = 0.720, respectively).

Patients who died within 30 days were significantly older than those who survived (79.8 ± 7.6 years vs. 76.2 ± 6.9 years, *p* < 0.001). The 30-day mortality group had a longer hospital stay and a higher rate of intensive care unit admission (*p* < 0.001 for both). There was no significant difference between the groups in glycemic control status or HbA1c levels. Regarding laboratory findings, uric acid, CRP, and ferritin levels were significantly higher in non-survivors, whereas HDL cholesterol and 25-hydroxyvitamin D levels were lower. Regarding disease severity scores, HALP scores were significantly lower in the death group, whereas EASIX and UHR scores were higher than in survivors (all *p* < 0.05) (Table 3).

In patients with poorly controlled blood glucose levels, univariate Cox regression analysis showed that advanced age, longer hospital stay, admission to the intensive care unit, higher uric acid, CRP, EASIX, and UHR scores, and lower HDL cholesterol and 25-hydroxyvitamin D levels were associated with a significant increase in 30-day mortality.

In multivariate analysis, age (HR: 1.066, 95% CI: 1.024–1.109, *p* = 0.002), length of hospital stay (HR: 1.050, 95% CI: 1.026–1.074, *p* < 0.001), admission to the intensive care unit (HR: 2.394, 95% CI: 1.227–4.672, *p* = 0.010), and UHR score (HR: 1.028, 95% CI: 1.013–1.042, *p* < 0.001) remained independent predictors of 30-day mortality (Table 4).

When patients were classified by their combined inflammation–nutrition indices, a gradual increase in 30-day mortality was observed across the higher-risk categories (Table 5).

In Model 1, patients with both high EASIX (>1.07) and UHR (≥12.93) had a significantly higher risk of 30-day mortality than the reference group, even after multivariate adjustment (adjusted HR: 1.062, 95% CI: 1.023–1.103, *p* = 0.020).

In Model 2, the co-occurrence of high EASIX and low HALP was independently associated with an increased risk of mortality (adjusted HR: 3.685, 95% CI: 1.650–8.229, *p* = 0.001).

Similarly, in Model 3, patients with high UHR and low HALP had the highest risk of death, with an adjusted HR of 4.206 (95% CI: 1.930–9.166, *p* < 0.001), representing more than a fourfold increase.

**Figure 2:**
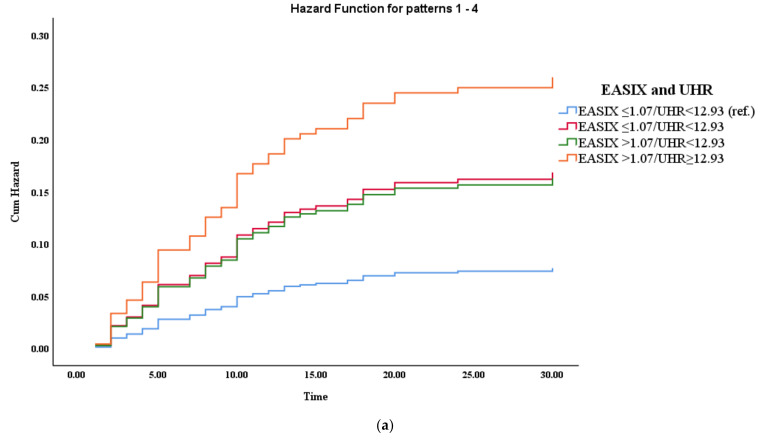

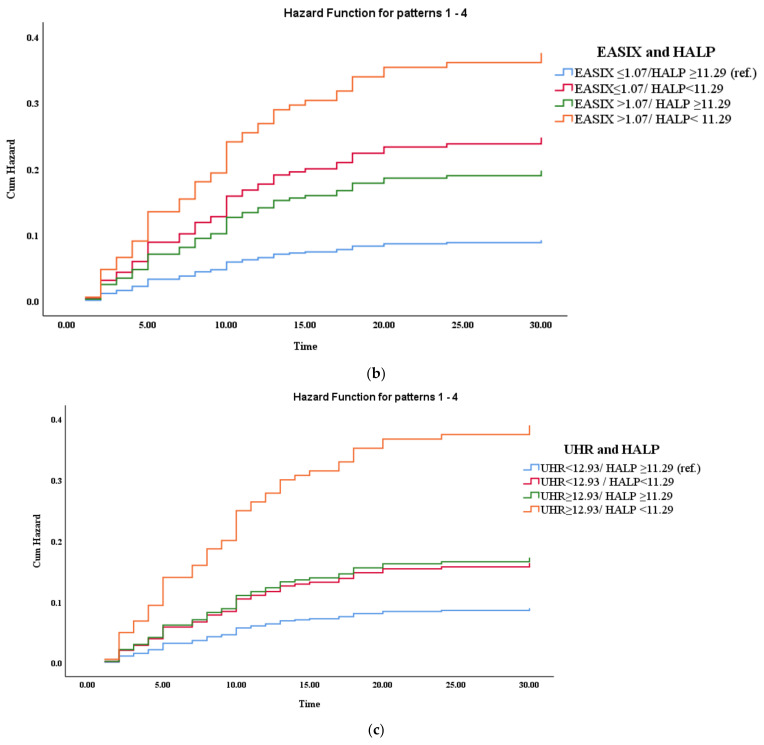
Cumulative hazard functions for 30-day mortality according to combined inflammatory and nutritional risk stratification. (**a**) Cumulative hazard of mortality stratified by combined EASIX and UHR categories. (**b**) Cumulative hazard of mortality stratified by combined EASIX and HALP categories. (**c**) Cumulative hazard of mortality stratified by combined UHR and HALP categories. Patients with concomitantly high inflammatory burden (EASIX > 1.07 and/or UHR ≥ 12.93) and poor nutritional status (HALP < 11.29) exhibited the highest cumulative hazard of 30-day mortality across all models.

Cumulative risk analyses revealed a clear and consistent trend in 30-day mortality across combined inflammatory and nutritional risk strata. A sharper and earlier increase in cumulative risk was observed in patients with concomitantly high inflammatory indices (high EASIX and/or UHR) and low HALP scores. Specifically, poor nutritional status significantly enhanced the prognostic effect of inflammatory burden, as reflected by the marked divergence of risk curves in the EASIX-HALP and UHR-HALP models (Figure 2).

## 4. Discussion

In this study, we investigated the relationship between glycemic control and inflammation–nutrition-based indices and their prognostic significance in older adults with type 2 diabetes. The main findings are as follows: (i) Poor glycemic control was associated with adverse inflammation–nutrition profiles, particularly higher HALP scores; (ii) Short-term mortality was not directly related to HbA1c levels but was strongly associated with inflammation burden and nutritional status; and (iii) Combined stratification using EASIX, UHR, and HALP provided superior risk discrimination for 30-day mortality compared with individual indices.

### Glycemic Control and Mortality

One of the most notable findings of this study is the lack of a significant association between HbA1c levels and 30-day mortality. Although HbA1c is widely accepted as an indicator of long-term glycemic control, it may not adequately reflect acute metabolic stress, inflammatory activation, and nutritional impairment during hospitalization, particularly in older adults. Acute illness-related factors such as systemic inflammation, endothelial dysfunction, and a catabolic state may overshadow the prognostic significance of chronic glycemic exposure in the short term. This finding is consistent with previous studies showing that HbA1c is a poor indicator of short-term mortality in elderly or critically ill diabetic patients [13,14].

In contrast, inflammation-based and nutritional parameters emerged as strong predictors of adverse outcomes. Patients who died within 30 days had higher CRP, ferritin, uric acid, EASIX, and UHR levels, along with lower HDL cholesterol, vitamin D, and HALP scores. These findings underscore the central role of chronic low-grade inflammation and impaired nutritional reserves in the pathophysiology of adverse outcomes in older adult diabetic patients. A high-inflammatory environment can exacerbate insulin resistance, promote endothelial dysfunction, and disrupt immune responses, ultimately increasing vulnerability during acute illness [15].

Among the disease severity indices assessed, HALP showed a distinct pattern, with significantly lower values in non-survivors. This result parallels findings by Zhu X. et al., who followed patients with type 2 diabetes mellitus who had acute ischemic stroke and found that low HALP scores were associated with worse functional outcomes and all-cause mortality [16]. However, results from the National Health and Nutrition Survey (NHANES) between 2005 and 2018 indicate an association between HALP scores and all-cause mortality and cardiovascular mortality among participants with diabetes or prediabetes [17].

HALP, a composite marker that includes hemoglobin, albumin, lymphocyte count, and platelet count, reflects both nutritional status and immune capacity. Low HALP scores likely indicate sarcopenia, hypoalbuminemia, anemia, and immune dysregulation; these are common features of frailty in older adults with diabetes. These factors can compromise physiological resistance and increase the risk of death by limiting the ability to recover from acute stressors.

The prognostic significance of EASIX and UHR further underscores the contribution of endothelial activation and metabolic–inflammatory load to mortality risk. EASIX, initially developed as an indicator of endothelial stress, was found to be significantly higher in patients with poor outcomes, suggesting a link between endothelial dysfunction and short-term mortality [18,19]. In line with the results of our study, Huang Y. et al. reported in their cohort study that EASIX is a reliable predictor of stroke prevalence and mortality [20]. Chen S. et al. reported that higher EASIX scores were associated with increased short-term mortality in critically ill patients with diabetic nephropathy. Similarly, in our study, UHR, which integrates uric acid and HDL cholesterol, emerged as an independent predictor of 30-day mortality in multivariate analysis. High uric acid can promote oxidative stress and inflammation, whereas low HDL levels impair anti-inflammatory and antioxidant defenses, thereby increasing cardiovascular and systemic risk [7,21,22]. A recent cross-sectional study in China shows a significant dose–response relationship between UHR and chronic kidney disease (CKD) in patients with type 2 diabetes, highlighting UHR as a promising biomarker for CKD risk assessment [23]. A cohort study of 17,968 participants aged 50 and older found that UHR was significantly associated with the incidence of age-related diseases and mortality, supporting our study [11].

Importantly, combined stratification models revealed a synergistic effect between inflammation and nutritional deficiency. Patients with concomitantly high EASIX or UHR and low HALP values consistently had the highest mortality across all models. This finding suggests that inflammation and malnutrition do not act in isolation but rather reinforce each other’s detrimental effects. From a clinical perspective, this interaction underscores the importance of assessing both inflammatory load and nutritional reserve when stratifying risk in older adults with diabetes.

Another clinically significant observation is that traditional comorbidities such as hypertension, coronary artery disease, and chronic renal failure are not independently associated with short-term mortality after adjustment. Instead, markers of acute disease severity, such as intensive care unit admission and prolonged hospital stay, along with inflammation–nutrition indices, better predict outcomes. This highlights that in older adult diabetic populations, dynamic physiological stressors and biological fragility may outweigh the influence of stable chronic conditions in determining short-term prognosis.

The findings of this study have important clinical implications. Inflammation–nutrition indices such as HALP, EASIX, and UHR are inexpensive, readily available, and easily derived from routine laboratory tests. Their combined use can facilitate early risk stratification, guide clinical decision-making, and identify high-risk patients who may benefit from closer monitoring, early nutritional support, and anti-inflammatory interventions.

Some limitations should be considered. The retrospective design limits causal inference, and residual confounding cannot be completely ruled out. The study was conducted at a single center, which may limit generalizability. Furthermore, dynamic changes in inflammatory and nutritional markers during hospitalization were not assessed. Despite these limitations, the relatively large sample size, comprehensive assessment of multiple inflammation–nutrition indices, and use of combined stratification models strengthen the validity of our findings.

## 5. Conclusions

In older adults with type 2 diabetes, short-term mortality appears to be more closely associated with inflammation, endothelial stress, and nutritional status than with glycemic control alone. Inflammation- and nutrition-based markers, particularly when used in combination, provide valuable prognostic information beyond HbA1c and may facilitate early risk stratification and more individualized management strategies in this vulnerable population. Future prospective, multicenter studies are warranted to validate these findings and to further evaluate the clinical utility of combined inflammation–nutrition indices in guiding risk stratification and clinical decision-making in older adults with type 2 diabetes.

## Figures and Tables

**Table 1:** Distribution of primary hospital admission diagnoses in older adults with diabetes mellitus.

Primary Reason for Hospital Admission	n = 372 (%)
Renal and Electrolyte Disorders	103 (27.7)
Endocrine and Metabolic Disorders	83 (22.3)
Anemia-Related Disorders	70 (18.8)
Infectious Diseases	49 (13.2)
Gastrointestinal Disorders	30 (8.1)
Cardiovascular Disorders	24 (6.5)
Respiratory Disorders	13 (3.5)

**Table 2:** Comparison of illness severity scores according to hemoglobin A1c levels (<7.5% vs. ≥7.5%).

Illness Severity Scores	HbA1c < 7.5% n = 215 (57.8%)	HbA1c ≥ 7.5% n = 157 (42.2%)	*p* Value
HALP, median (IQR)	18.58 (11.81–31.55)	26.87 (14.75–42.58)	<0.001
EASIX, median (IQR)	0.93 (0.50–1.70)	0.83 (0.58–1.36)	0.566
UHR, median (IQR)	14.69 (9.55–21.11)	14.05 (10.00–19.41)	0.720

EASIX, Endothelial Activation and Stress Index; HALP, Hemoglobin, Albumin, Lymphocyte, Platelet Score; UHR, uric acid-to-high-density cholesterol ratio.

**Table 3:** Baseline characteristics according to 30-day mortality.

Parameters	Survivors n = 315 (84.7%)	30-Day Mortality, n = 57 (15.3%)	*p* Value
**Demographic and Clinical Characteristics**			
Sex (Male/Female), n (%)	98 (31.1)	25 (43.9)	0.060
Age (year), mean ± SD	76.2 ± 6.9	79.8 ± 7.6	<0.001
Glycemic Control Status (Good/Poor), n (%)	180/135 (57.1/42.9)	35/22 (61.4/38.6)	0.549
Mean arterial pressure (mmHg), mean ± SD	91.1 ± 13.7	90.6 ± 14.7	0.808
Hypertension, n (%)	268 (85.1)	46 (80.7)	0.402
Chronic renal failure, n (%)	42 (13.3)	8 (14.0)	0.886
Coronary artery disease, n (%)	148 (47.0)	30 (52.6)	0.432
Stroke history, n (%)	37 (11.7)	10 (17.5)	0.225
Number of chronic medications, mean ± SD	6.3 ± 2.8	6.20 ± 2.6	0.913
Length of hospital stay (days), median (IQR)	6 (4.0–8.5)	9.0 (5.0–15.0)	<0.001
ICU admission, n (%)	28 (8.9)	15 (26.3)	<0.001
**Laboratory Parameters**			
Hemoglobin (g/dL), mean ± SD	10.6 ± 1.9	10.5 ± 1.9	0.785
Hemoglobin A1c (%), median (IQR)	7.2 (6.3–8.8)	7.0 (6.4–8.5)	0.615
eGFR (mL/dk/1.73 m^2^), mean ± SD	57.7 ± 24.8	51.2 ± 28.2	0.078
Glucose (mg/dL), median (IQR)	138.0 (104.0–192.0)	123.0 (96.0–182.0)	0.318
Uric Acid (mg/dL), mean ± SD	5.9 ± 2.4	7.0 ± 3.1	0.019
Creatinine (mg/dL), median (IQR)	1.06 (0.80–1.47)	1.28 (0.90–1.80)	0.038
Lactate dehydrogenase (U/L), median (IQR)	195.0 (161.0–239.5)	227.0 (179.0–313.0)	0.003
Total Cholesterol (mg/dL), mean ± SD	161.7 ± 49.9	154.3 ± 49.2	0.314
LDL (mg/dL), mean ± SD	93.0 ± 41.5	89.5 ± 38.0	0.567
HDL (mg/dL), mean ± SD	42.6 ± 14.6	36.6 ± 15.1	0.001
Platelet count (×10^3^/µL) median (IQR)	257.0 (189.5–334.5)	233.0 (166.0–287.0)	0.067
Lymphocyte count (×10^3^/µL), median (IQR)	1.55 (1.10–2.21)	1.17 (0.8–1.6)	0.001
Triglyceride (mg/dL), median (IQR)	137.0 (98.0–186.0)	122.0 (82.0–188.0)	0.339
C-reactive protein (mg/L), median (IQR)	11.7 (2.9–41.0)	37.0 (13.0–89.9)	<0.001
Albumin (g/dL), mean ± SD	3.7 (3.2–4.0)	3.1 (2.7–3.8)	<0.001
Ferritin (µg/L), median (IQR)	115.0 (41.0–285.0)	201.0 (70.0–431.5)	0.011
Vitamin B12 (ng/L), mean ± SD	653.6 ± 509.8	755.8 ± 579.9	0.191
25-Hydroxyvitamin D (µg/L), mean ± SD	18.2 ± 11.6	14.3 ± 10.3	0.023
**Illness severity scores**			
HALP score, median (IQR)	21.98 (13.68–36.76)	16.94 (9.49–29.28)	0.010
EASIX score, median (IQR)	0.84 (0.51–1.46)	1.23 (0.68–2.81)	0.001
UHR score, median (IQR)	13.82 (9.45–19.40)	16.92 (11.92–30.45)	0.002

EASIX, endothelial activation and stress index; eGFR, estimated glomerular filtration rate; HALP, hemoglobin, albumin, lymphocyte, platelet score; HDL, high density lipoprotein; ICU, intensive care unit; IQR, interquartile range; LDL, low-density lipoprotein; SD, standard deviation; UHR, uric acid-to-high-density cholesterol ratio.

**Table 4:** Cox regression analysis identifying predictors of 30-day mortality in patients with poor glycemic control.

	Univariate Analysis				Multivariate Analysis			
	HR (95% CI)	*p* Value	Wald	β	HR (95% CI)	*p* Value	Wald	β
Sex	1.679 (0.995–2.833)	0.052	3.768	0.518				
Age (years)	1.068 (1.031–1.107)	<0.001	13.253	0.066	1.066 (1.024–1.109)	0.002	9.824	0.064
Length of hospital stay (days)	1.048 (1.029–1.067)	<0.001	25.164	0.047	1.050 (1.026–1.074)	<0.001	17.077	0.048
ICU admission	3.133 (1.737–5.653)	<0.001	14.391	1.142	2.394 (1.227–4.672)	0.010	10.454	1.312
eGFR (mL/min/1.73 m^2^)	0.990 (0.980–1.001)	0.079	3.092	−0.010				
Uric Acid (mg/dL)	1.146 (1.048–1.254)	0.003	8.891	0.137	Excluded			
HDL cholesterol (mg/dL)	0.967 (0.948–0.986)	0.001	11.232	−0.034				
C-reactive protein (mg/L)	1.006 (1.002–1.009)	0.002	9.702	0.006				
Ferritin (µg/L)	1.001 (1.000–1.001)	0.055	3.675	0.001				
25-Hydroxyvitamin D (µg/L)	0.968 (0.942–0.996)	0.025	5.053	−0.032				
HALP score	0.986 (0.970–1.001)	0.073	3.209	−0.014				
EASIX score	1.088 (1.030–1.150)	0.003	8.990	0.085				
UHR score	1.045 (1.024–1.067)	<0.001	25.885	0.033	1.028 (1.013–1.042)	<0.001	14.236	0.027

CI, confidence interval; EASIX, endothelial activation and stress index; eGFR, estimated glomerular filtration rate; HALP, hemoglobin, albumin, lymphocyte, platelet score; HDL, high density lipoprotein; HR, hazard ratio; ICU, intensive care unit; UHR, uric acid-to-high-density cholesterol ratio.

**Table 5:** Multivariable cox regression analysis of 30-day mortality based on combined EASIX, UHR, and HALP stratification.

		Hazard Ratio (95% CI)			
EASIX/UHR (Model 1)	Deaths/Total (%)	Crude HR (95% CI)	*p*Value	Adjusted HR (95% CI)	*p* Value
EASIX ≤ 1.07/UHR < 12.93	8/108 (7.4)	Reference	-	Reference	-
EASIX ≤ 1.07/UHR ≥ 12.93	16/103 (15.5)	2.192 (0.938–5.123)	0.070	2.112 (0.904–4.937)	0.084
EASIX > 1.07/UHR < 12.93	7/47 (14.9)	2.119 (0.768–5.843)	0.147	1.850 (0.669–5.116)	0.236
EASIX > 1.07/UHR ≥ 12.93	26/114 (22.8)	3.380 (1.530–7.467)	0.003	1.062 (1.023–1.103)	0.020
EASIX/HALP (Model 2)					
EASIX ≤ 1.07/HALP ≥ 11.29	15/170 (8.8)	Reference	-	Reference	-
EASIX ≤ 1.07/HALP < 11.29	9/41 (21.9)	2.686 (1.175–6.140)	0.019	2.639 (1.150–6.052)	0.022
EASIX > 1.07/HALP ≥11.29	23/129 (17.8)	2.143 (1.118–4.108)	0.022	1.664 (0.857–3.233)	0.133
EASIX > 1.07/HALP < 11.29	10/32 (31.3)	4.074 (1.829–9.072)	0.001	3.685 (1.650–8.229)	0.001
UHR/HALP (Model 3)					
UHR < 12.93/HALP ≥ 11.29	11/128 (8.5)	Reference	-	Reference	-
UHR < 12.93/HALP < 11.29	4/27 (14.8)	1.826 (0.581–5.734)	0.303	1.735 (0.549–5.484)	0.348
UHR ≥ 12.93/HALP ≥ 11.29	27/171 (15.8)	1.924 (0.954–3.879)	0.067	1.626 (0.802–3.296)	0.177
UHR ≥ 12.93/HALP < 11.29	15/46 (32.6)	4.341 (1.993–9.455)	<0.001	4.206 (1.930–9.166)	<0.001

CI, confidence interval; EASIX, endothelial activation and stress index; HALP, hemoglobin, albumin, lymphocyte, platelet score; HR, hazard ratio; UHR, uric acid-to-high-density cholesterol ratio.

## Data Availability

The datasets used and/or analyzed during the current study are available from the corresponding author upon reasonable request.

## Abbreviations

The following abbreviations are used in this manuscript:

EASIX	Endothelial activation and stress index
HALP	Hemoglobin, albumin, lymphocyte, platelet score
UHR	Uric acid-to-high-density cholesterol ratio
